# Evaluation of the Cost-effectiveness of Infection Control Strategies to Reduce Hospital-Onset *Clostridioides difficile* Infection

**DOI:** 10.1001/jamanetworkopen.2020.12522

**Published:** 2020-08-13

**Authors:** Anna K. Barker, Elizabeth Scaria, Nasia Safdar, Oguzhan Alagoz

**Affiliations:** 1 Department of Internal Medicine, University of Michigan, Ann Arbor; 2 Department of Population Health Sciences, School of Medicine and Public Health, University of Wisconsin–Madison; 3 Department of Industrial and Systems Engineering, College of Engineering, University of Wisconsin–Madison; 4 Division of Infectious Diseases, Department of Medicine, School of Medicine and Public Health, University of Wisconsin–Madison; 5 William S. Middleton Memorial Veterans Hospital, Madison, Wisconsin

## Abstract

**Importance:**

*Clostridioides difficile* infection is the most common hospital-acquired infection in the United States, yet few studies have evaluated the cost-effectiveness of infection control initiatives targeting *C difficile*.

**Objective:**

To compare the cost-effectiveness of 9 *C difficile* single intervention strategies and 8 multi-intervention bundles.

**Design, Setting, and Participants:**

This economic evaluation was conducted in a simulated 200-bed tertiary, acute care, adult hospital. The study relied on clinical outcomes from a published agent-based simulation model of *C difficile* transmission. The model included 4 agent types (ie, patients, nurses, physicians, and visitors). Cost and utility estimates were derived from the literature.

**Interventions:**

Daily sporicidal cleaning, terminal sporicidal cleaning, health care worker hand hygiene, patient hand hygiene, visitor hand hygiene, health care worker contact precautions, visitor contact precautions, *C difficile* screening at admission, and reduced intrahospital patient transfers.

**Main Outcomes and Measures:**

Cost-effectiveness was evaluated from the hospital perspective and defined by 2 measures: cost per hospital-onset *C difficile* infection averted and cost per quality-adjusted life-year (QALY).

**Results:**

In this agent-based model of a simulated 200-bed tertiary, acute care, adult hospital, 5 of 9 single intervention strategies were dominant, reducing cost, increasing QALYs, and averting hospital-onset *C difficile* infection compared with baseline standard hospital practices. They were daily cleaning (most cost-effective, saving $358 268 and 36.8 QALYs annually), health care worker hand hygiene, patient hand hygiene, terminal cleaning, and reducing intrahospital patient transfers. Screening at admission cost $1283/QALY, while health care worker contact precautions and visitor hand hygiene interventions cost $123 264/QALY and $5 730 987/QALY, respectively. Visitor contact precautions was dominated, with increased cost and decreased QALYs. Adding screening, health care worker hand hygiene, and patient hand hygiene sequentially to the daily cleaning intervention formed 2-pronged, 3-pronged, and 4-pronged multi-intervention bundles that cost an additional $29 616/QALY, $50 196/QALY, and $146 792/QALY, respectively.

**Conclusions and Relevance:**

The findings of this study suggest that institutions should seek to streamline their infection control initiatives and prioritize a smaller number of highly cost-effective interventions. Daily sporicidal cleaning was among several cost-saving strategies that could be prioritized over minimally effective, costly strategies, such as visitor contact precautions.

## Key Points

**Question:**

What is the most cost-effective infection control strategy for reducing hospital-onset *Clostridioides difficile* infection?

**Findings:**

In this economic evaluation study, an agent-based simulation of *C difficile* transmission at a 200-bed model hospital found 5 dominant interventions that reduced costs and improved outcomes compared with baseline practices, as follows: daily cleaning (the most cost-effective, saving $358 268 and 36.8 quality-adjusted life-years annually), terminal cleaning, health care worker hand hygiene, patient hand hygiene, and reduced intrahospital patient transfers. The incremental cost-effectiveness of implementing multiple intervention strategies quickly decreased beyond a 2-pronged bundle.

**Meaning:**

The findings of this study suggest that institutions should streamline infection control bundles, prioritizing a small number of highly cost-effective interventions.

## Teaser

This economic evaluation study compares the cost-effectiveness of 9 *Clostridioides difficile *single intervention strategies and 8 multi-intervention bundles.

## Introduction

*Clostridioides difficile *is the most common hospital-acquired infection in the United States, responsible for more than 15 000 deaths and $5 billion in direct health care costs annually.^1^ Health care facilities are a major source of new infections, and in-hospital prevention is critical to decreasing its overall incidence. Efforts to control *C difficile* infection (CDI) have intensified in recent years, with the addition of CDI to Medicare’s Hospital-Acquired Condition Reduction Program.^2^ However, the results of targeted infection control initiatives have been variable, and CDI incidence continues to rise.^1,3,4^

Nationwide, interventions are typically implemented simultaneously in multi-intervention bundles.^3^ This strategy makes it impossible to identify the isolated effects of single interventions using traditional epidemiologic methods.^5^ However, by developing an agent-based simulation model of *C difficile* transmission, our group was previously able to evaluate the clinical effectiveness of 9 interventions and 8 multi-intervention bundles in a simulated general, 200-bed, adult hospital.^6^ All hospitals operate in a setting of constrained resources. Thus, evaluating the cost-effectiveness of common infection control interventions is essential to providing evidence-based recommendations regarding which strategies to prioritize and implement.

While several *C difficile* cost-effectiveness studies have been published, the overwhelming majority focus on comparing treatment or diagnostic testing modalities.^7^ Among those that assess infection control initiatives, most evaluate a single intervention or single bundle. To our knowledge, only 2 other studies^8,9^ have investigated the comparative cost-effectiveness of multiple *C difficile* interventions. Neither evaluated emerging patient-centered interventions, such as screening at admission or patient hand hygiene. Furthermore, both studied environmental cleaning only as a bundled strategy and did not distinguish between daily and terminal cleaning^8^ or daily cleaning, terminal cleaning, and hand hygiene.^9^ Daily cleaning and screening are highly effective in their own right,^6,10,11^ and an evaluation of the cost-effectiveness of single-intervention strategies such as these is essential. Thus, we aimed to evaluate the cost-effectiveness of 9 infection control interventions and 8 multi-intervention bundles using an agent-based model of adult *C difficile* transmission.

## Methods

### Approach

We previously published an agent-based model of *C difficile* transmission in a simulated general, 200-bed, tertiary, acute care adult hospital.^6^ Output from this model was used to evaluate the cost-effectiveness of infection control strategies in terms of 2 primary outcomes: the cost per quality-adjusted life-year (QALY) saved and cost per hospital-onset CDI (HO-CDI) averted. The study was reviewed and approved by the University of Wisconsin–Madison institutional review board. This study follows the recommendations of the Consolidated Health Economic Evaluation Reporting Standards (CHEERS) reporting guideline.^12^

### Agent-Based Model

For additional modeling details, see the eAppendix in the Supplement. Briefly, the model simulated a dynamic hospital environment and 4 agent types (ie, patients, visitors, nurses, and physicians), during a 1-year time period (eFigure 1 in the Supplement).^6^ Patients were categorized into 1 of 9 clinical states representing their CDI-related status. These clinical states were updated every 6 hours by a discrete-time Markov chain. Patients in the colonized, infected, recolonized, or recurrent infection states were contagious and could transmit *C difficile* to other agents and the environment. Once contaminated, visitors, nurses, physicians, and the environment could transmit *C difficile* to susceptible patients and the environment. The probability of transmission occurring during a given interaction was dependent on the agent types involved and the duration of the interaction (eTable 1 in the Supplement). Key model parameter estimates are shown in Table 1.^6,10,13,14,15,16,17,18,19,20,21,22,23,24,25,26,27,28,29,30,31,32,33,34,35,36,37,38,39,40,41,42,43,44,45,46,47,48,49,50,51,52,53,54,55,56,57,58,59,60,61,62,63,64,65,66,67,68,69,70,71,72,73,74,75,76,77,78,79,80,81,82,83,84,85,86,87,88,89,90,91,92,93,94^ The model was developed and run in NetLogo software version 5.3.1.^95^ We used synchronized random numbers, which allowed us to directly compare runs under different intervention scenarios, while minimizing variability owing to chance.^96^

**Table 1.** Select Parameter Estimates for the Agent-Based Model

Admission parameter	Mean, %			Source
	Baseline	Enhanced	Ideal	
Patient length of stay, mean (SD), d	4.8 (4.8)	4.8 (4.8)	4.8 (4.8)	AHA,^13^ 2016; AHRQ,^14^ 2012; AHRQ,^15^ 2012; Kaboli et al,^16^ 2012
Proportion in each category at admission (total 100%)				
Susceptible patients	39.7	39.7	39.7	AHRQ,^14^ 2012; CDC,^17^ 2010; Hicks et al,^18^ 2015; Frenk et al,^19^ 2016; Dantes et al,^20^ 2015
Asymptomatic colonized	6.1	6.1	6.1	Longtin et al,^10^ 2016; Koo et al,^21^ 2014; Alasmari et al,^22^ 2014; Leekha et al,^23^ 2013; Loo et al,^24^ 2011; Eyre et al,^25^ 2013; Nissle et al,^26^ 2016; Kagan et al,^27^ 2017; Gupta et al,^28^ 2012; Hung et al,^29^ 2013; Dubberke et al,^30^ 2015
Patients with *C difficile *infection	0.29	0.29	0.29	Koo et al,^21^ 2014; Kagan et al,^27^ 2017; AHRQ,^31^ 2009; Evans et al,^32^ 2014
Nonsusceptible patients	53.9	53.9	53.9	NA
Hand hygiene				
Effectiveness at spore removal				
Soap and water	96	96	96	Bettin et al,^33^ 1994; Oughton et al,^34^ 2009; Edmonds et al,^35^ 2013; Jabbar et al,^36^ 2010
ABHR	29	29	29	
Compliance in standard room				
Nurse	60	79	96	Dierssen-Sotos et al,^37^ 2010; Randle et al,^38^ 2013; Monistrol et al,^39^ 2012; Tromp et al,^40^ 2012; Kowitt et al,^41^ 2013; Mestre et al,^42^ 2012; Eldridge et al,^43^ 2006; Zerr et al,^44^ 2005; Mayer et al,^45^ 2011; Muto et al,^46^ 2007; Grant and Hofmann,^47^ 2011; Grayson et al,^48^ 2011; Pittet et al,^49^ 2004; Clock et al,^50^ 2010; Birnbach et al,^51^ 2015; Randle et al,^52^ 2014; Birnbach et al,^53^ 2012; Caroe Aarestrup et al,^54^ 2016; Nishimura et al,^55^ 1999; Randle et al,^56^ 2010; Davis,^57^ 2010; Srigley et al,^58^ 2014; Cheng et al,^59^ 2007; Hedin et al,^60^ 2012; Gagné et al,^61^ 2010
Doctor	50	71	91	
Visitor	35	55	84	
Patient	33	59	84	
Fraction of soap and water vs ABHR use in standard room	10	10	10	Mestre et al,^42^ 2012; Stone et al,^62^ 2007
Compliance in known *C difficile* room^a^				Golan et al,^63^ 2006; Morgan et al,^64^ 2013; Swoboda et al,^65^ 2007; Almaguer-Leyva et al,^66^ 2013
Nurse	69	84	97	
Doctor	61	77	93	
Visitor	50	65	88	
Patient	48	68	88	
Fraction soap and water vs ABHR use in known *C difficile* room	80	90	95	Zellmer et al,^67^ 2015
Contact precautions				
Gown and glove effectiveness at preventing spore contamination	70	86	97	Morgan et al,^68^ 2012; Landelle et al,^69^ 2014; Tomas et al,^70^ 2015
Health care worker compliance	67	77	87	Clock et al,^50^ 2010; Morgan et al,^64^ 2013; Weber et al,^71^ 2007; Manian and Ponzillo,^72^ 2007; Bearman et al,^73^ 2007; Bearman et al,^74^ 2010; Deyneko et al,^75^ 2016
Visitor compliance	50	74	94	Clock et al,^50^ 2010; Weber et al,^71^ 2007; Manian and Ponzillo,^72^ 2007
Environmental cleaning				
Daily cleaning compliance	46	80	94	Sitzlar et al,^76^ 2013; Goodman et al,^77^ 2008; Hayden et al,^78^ 2006; Boyce et al,^79^ 2009
Terminal cleaning compliance	47	77	98	Sitzlar et al,^76^ 2013; Hess et al,^80^ 2013; Ramphal et al,^81^ 2014; Anderson et al,^82^ 2017; Clifford et al,^83^ 2016; Carling et al,^84^ 2008
Nonsporicidal effectiveness at spore removal	45	45	45	Nerandzic and Donskey,^85^ 2016; Wullt et al,^86^ 2003
Sporicidal effectiveness at spore removal	99.6	99.6	99.6	Wullt et al,^86^ 2003; Perez et al,^87^ 2005; Deshpande et al,^88^ 2014; Block et al,^89^ 2004
Screening				
Compliance	0	96	98	Jain et al,^90^ 2001; Harbath et al,^91^ 2008
PCR test				
Sensitivity	93	93	93	Deshpande et al,^92^ 2011; Bagdasarian et al,^93^ 2015; O’Horo et al,^94^ 2012
Specificity	97	97	97	
Patient transfer rate				
Intraward	5.7	2.8	1.4	ID
Interward	13.7	6.8	3.4	

Abbreviations: ABHR, alcohol-based hand rub; ID, internal data; NA, not applicable; PCR, polymerase chain reaction.

^a^ Based on standard room estimates and standard-to-known *C difficile* room hand hygiene noncompliance ratio of 1.34, adapted from Barker et al.^6^

### Interventions

We simulated the effects of 9 interventions, as follows: daily cleaning with sporicidal products; terminal cleaning with sporicidal products; patient hand hygiene; visitor hand hygiene; health care worker hand hygiene; visitor contact precautions; health care worker contact precautions; reduced intrahospital patient transfers; and screening for asymptomatic *C difficile* colonization at admission. Each intervention was modeled individually at an enhanced and ideal implementation level that reflected typical and optimal implementation contexts, respectively. We also simulated 8 infection control bundles that included between 2 and 5 enhanced-level interventions. Ideal-level interventions were not included in the bundle strategies because in general they did not result in considerable improvement compared with enhanced-level strategies. Thus, they were not deemed a high priority for bundle inclusion.

All strategies were compared with a baseline state, in which no interventions were enacted but standard hospital practices, such as hand hygiene, occurred at rates expected in a nonintervention context (Table 1). Ideal-level single interventions were also compared with the enhanced-level of each intervention, and bundles were compared among themselves. Each single intervention and bundle was simulated 5000 times. One replication of the simulation took approximately 115 seconds on a single core of a 1.80 GHz Intel Core i5-5350U processor with 8 GB of RAM running macOS Mojave version 10.14.3.

### Cost

This study was conducted from the hospital perspective. Cost estimates (Table 2^1,14,62,97,98,99,100,101,102,103,104,105,106,107,108,109,110,111,112,113,114,115,116,117,118,119,120,121,122,123,124,125,126,127,128,129,130,131,132,133,134,135,136,137,138,139,140^) were derived from the literature and converted into 2018 US dollars using the Personal Consumption Expenditure Health Index.^141^ Fixed and variable costs were considered. Both were higher for corresponding ideal-level vs enhanced-level interventions. Fixed costs included the cost of additional infection control staffing to implement, support, and serially evaluate compliance with an intervention (eAppendix in the Supplement). Ideal-level interventions had increased intervention compliance. Thus, the variable costs inherent in each successful intervention event (ie, alcohol-based hand rub product, labor related to alcohol-based hand rub hygiene time) also increased. We assumed that all costs occurred in the same year as the patient’s hospital visit; therefore, costs were not discounted. The excess cost attributable to a single CDI was estimated at $12 313 (range, $6156-$18 469).^100,102,142^

**Table 2.** Infection and Infection Control–Related Cost and QALY Estimates

Parameter	Mean (range), 2018 US $	Source
**Fixed costs**		
Standard education and printing materials	1535 (556-2386)	Nelson et al,^97^ 2016; Nyman et al,^98^ 2011; Stone et al,^62^ 2007
Education and printing materials for serial campaigns^a^	4606 (1669-7157)	Nelson et al,^97^ 2016; Nyman et al,^98^ 2011; Stone et al,^62^ 2007
Full-time infection preventionist salary and benefits^b^	111 527 (94 798-128 256)	Nelson et al,^97^ 2016; Nyman et al,^98^ 2011; BLS,^99^ 2019
PCR laboratory equipment annual overhead cost for screening	5563 (5007-6120)	Nyman et al,^98^ 2011
**Variable costs**		
General		
Excess hospital cost attributable to *C difficile* infection	12 313 (6156-18 469)	Zimlichman et al,^100^ 2013; AHRQ,^101^ 2017; Magee et al,^102^ 2015
Physician hourly wage and benefits, mean^c^	115.34	BLS,^99^ 2019
Nurse hourly wage and benefits, mean^c^	48.58	
Cleaning staff hourly wage and benefits, mean^c^	18.56	
Hand hygiene		
Soap and water labor time, s	23 (15-40)	Cimiotti et al,^103^ 2004; Larson et al,^104^ 2001; Voss and Widmer,^105^ 1997; Girou et al,^106^ 2002
Soap and water product	0.06 (0.03-0.10)	Stone et al,^62^ 2007; Larson et al,^104^ 2001; Boyce,^107^ 2001
ABHR labor time, s	13 (5-20)	Cimiotti et al,^103^ 2004; Larson et al,^104^ 2001; Voss and Widmer,^105^ 1997; Girou et al,^106^ 2002
ABHR product	0.03 (0.02-0.04)	Stone et al,^62^ 2007; Larson et al,^104^ 2001
Contact precautions		
Donning and doffing labor time, s	60 (35-95)	Puzniak et al,^108^ 2004; Papia et al,^109^ 1999
Gloves product	0.09 (0.12-0.15)	
Gown product	0.75 (0.49-1.01)	
Environmental cleaning		
UV light and fluorescent gel to assess compliance	435 (200-500)	Glogerm^110^; Glitterbug^111^; CDC,^112^ 2010; ID
Standard daily cleaning supplies per room^d^	0.91 (0.68-1.14)	Saha et al,^113^ 2016; ID
Standard terminal cleaning supplies per room^d^	1.34 (1.00-1.67)	
Sporicidal daily cleaning supplies per room^e^	1.05 (0.79-1.32)	
Sporicidal terminal cleaning supplies per room^e^	2.19 (1.65-2.74)	
Daily cleaning staff labor time, min	15 (10-20)	Doan et al,^114^ 2012; ASHES,^115^ 2009; ID
Terminal cleaning staff labor time, min	50 (40-60)	
Screening		
PCR test materials	6.99 (3.69-17.67)	Curry et al,^116^ 2011; Schroeder et al,^117^ 2014
Overhead on testing supplies, eg, delivery, storage, %	20	Nyman et al,^98^ 2011
Labor collection time per swab, min	5 (3-7)	Nyman et al,^98^ 2011
Nursing assistant hourly wage and benefits^c^	19.72	BLS,^99^ 2019
Laboratory technician time, min	14 (10-25)	Nyman et al,^98^ 2011; Curry et al,^116^ 2011; Schroeder et al,^117^ 2014; Sewell et al,^140^ 2014
Laboratory technician hourly wage and benefits, mean^c^	34.83	BLS,^99^ 2019
Patient transfer^f^		
Transport staff intraward transport labor time, min	7 (5-15)	Hendrich and Lee,^118^ 2005
Transport staff interward transport labor time, min	15 (7-25)	Hendrich and Lee,^118^ 2005
Transport staff hourly wage and benefits, mean	18.84	BLS,^99^ 2019
Handoff time, per nurse in interward transfers only, min	10 (5-15)	Hendrich and Lee,^118^ 2005; Catchpole et al,^119^ 2007; Rayo et al,^120^ 2014
**QALY-related estimates**		
Utilities		
Age of healthy patients, y		
18-34	0.91	Gold et al,^121^ 1998; Swinburn and Davis,^122^ 2013
35-64	0.88	
65-84	0.85	
≥85	0.83	
* C difficile* infection	0.81 (0.70-0.86)	Ramsey et al,^123^ 2005; Bartsch et al,^124^ 2012; Konijeti et al,^125^ 2014; Tsai et al,^126^ 2008; Thuresson et al,^127^ 2011
Age of all hospitalized patients, y		
18-34	14.8	AHRQ,^14^ 2012; AHRQ,^128^ 2010
35-64	43.8	
65-84	31.7	
≥85	9.7	
Age of patients with CDI, %		
18-34 y	5.7	AHRQ,^129^ 2019; IDPH,^130^ 2019
35-64 y	31.7	
65-84 y	44.6	
≥85 y	18.0	
Life expectancy by age, y^g^		
25	54.9	National Vital Statistics Report,^131^ 2014
50	31.7	
75	12.3	
≥85	6.7	
Mean Charlson Comorbidity Index score for in-hospital CDI patients	2.57	Magee et al,^102^ 2015
QALYs lost owing to CDI-related mortality by age, No.^h^		
26 y	48.11 (36.14-60.24)	NA
49.5 y	28.70 (22.04-36.73)	
74.5 y	12.00 (9.13-15.21)	
85 y	6.39 (4.98-8.30)	
**Other**		
Time at lower utility owing to symptomatic diarrhea, d	4.2 (3.15-5.25)	Sethi et al,^132^ 2010; Bobulsky et al,^133^ 2008
Hospitalization utility value	0.63	Shaw et al,^134^ 2005
Proportion of modeled deaths among CDI patients attributable to CDI	0.48	Tabak et al,^135^ 2013; Lessa et al,^1^ 2015
Proportion of patients with CDI readmitted within 30 d, %	23.2 (20.0-30.1)	Magee et al,^102^ 2015; Chopra et al,^136^ 2015; AHRQ,^137^ 2009
Proportion of patients with no CDI readmitted within 30 d, %	14.4 (13.9-14.8)	Magee et al,^102^ 2015; Chopra et al,^136^ 2015; AHRQ,^138^ 2013; AHRQ,^139^ 2014

Abbreviations: ABHR, alcohol-based hand rub; AHRQ, Agency for Healthcare Research and Quality; ASHES, American Society for Healthcare Environmental Services; BLS, Bureau of Labor Statistics; CDC, US Centers for Disease Control and Prevention; CDI, *Clostridioides difficile* infection; IDPH, Illinois Department of Public Health; NA, not applicable; PCR, polymerase chain reaction; QALY, quality-adjusted life year; UV, ultraviolet.

^a^ Enhanced health care worker, patient, and visitor hand hygiene and health care worker and visitor contact precautions as well as all ideal-level campaigns.

^b^ For details regarding intervention specific staffing requirements, see the Cost subsection in Methods.

^c^ These data are based on BLS data; no range is available.

^d^ Category includes nonsporicidal quaternary ammonium solution, mops, and rags.

^e^ Category includes peracetic acid and/or hydrogen peroxide solution, mops, and rags.

^f^ Each patient transfer also requires an additional terminal cleaning per patient hospitalization.

^g^ Parameterizes time horizon.

^h^ Data in this section was based on calculations from Table 1.

### Outcomes

The number of HO-CDIs per year was output directly from the model for each run.^6^ We defined HO-CDI based on the Centers for Disease Control and Prevention’s guidelines as symptomatic diarrhea plus a positive laboratory test result on a specimen collected more than 3 days after hospital admission.^143^ We calculated QALYs using model output and the utility values shown in Table 2. To determine the QALYs lost because of CDI-associated mortality, the age distribution for CDI cases was used in conjunction with age-specific utility values from healthy adults. Mean life expectancies were derived from the Centers for Disease Control and Prevention life tables, accounting for a mean Charlson Comorbidity Index for in-hospital CDI patients of 2.57.^102^ The total number of deaths output from the model was multiplied by 0.48 to account for *C difficile*–associated mortality.^1,135^ Discounting future QALYs is controversial^144^; thus, they were not discounted in the primary analysis, similar to costs. Results of a supplemental analysis in which future QALYs were discounted at 3% is included in eTable 2 in the Supplement.

The minor loss in QALYs due to CDI symptoms was calculated from a mean symptomatic period of 4.2 days and utility value for symptomatic CDI of 0.81.^132,133^ Since there is no established utility measure of CDI in the United States, this followed a standard practice of basing it on that of noninfectious diarrhea.^123,124,125,126,127^ A loss in QALYs owing to time spent in a hospital admission was accounted for with a 0.63 utility value for hospitalized patients, derived using the EuroQol-5D instrument.^134^ Thus, it was possible to have a net negative QALY, despite a minimally net positive CDI averted.

### Statistical Analysis

Incremental cost-effectiveness ratios (ICERs) for HO-CDIs averted and QALYs gained were calculated using 2 methods. In the first approach, we found means for each intervention’s costs, HO-CDIs, and QALYs across all runs. We then calculated ICERs using these means for compared interventions. In the second method, an ICER was calculated based on the costs, HO-CDIs, and QALYs of 2 interventions for each run. These ICERs were then used to calculate the proportion of runs that met 21 willingness-to-pay thresholds. We assumed that any run resulting in negative incremental QALYs was not cost-effective. Analysis was conducted in R version 3.4.3 (R Project for Statistical Computing). No statistical testing was performed, so no prespecified level of significance was set.

A probabilistic sensitivity analysis was conducted varying cost and QALY parameter estimates simultaneously. Estimates were varied using the triangular distribution, with the minimum, mean, and maximum values reported in Table 2. Each single intervention and bundle simulation was run 100 000 times. One-way sensitivity analyses were also performed using the minimum and maximum reported values (Table 2).

## Results

**Figure 1.**
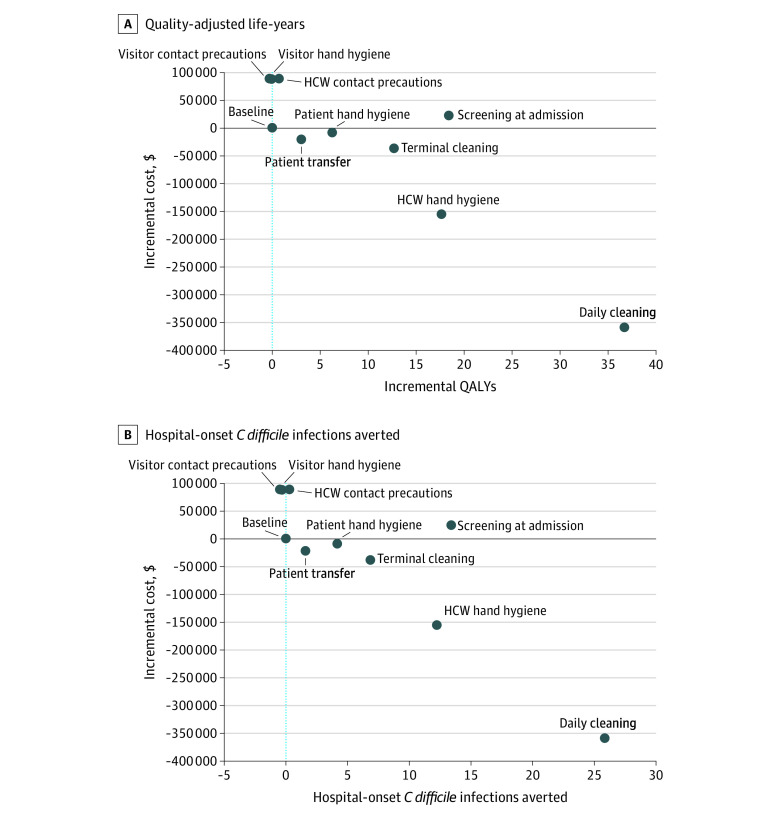
Incremental Cost vs Quality-Adjusted Life-Years (QALYs) and Hospital-Onset *Clostridioides difficile *Infections Averted for Enhanced Interventions, Compared With Baseline HCW indicates health care worker.

In this agent-based model of a simulated 200-bed tertiary, acute care, adult hospital, 5 of 9 enhanced-level interventions were dominant compared with baseline hospital practices, resulting in cost savings, increased QALYs, and averted infections, as follows: daily cleaning (the most cost-effective, saving $358 268, 25.9 infections, and 36.8 QALYs annually), terminal cleaning, health care worker hand hygiene, patient hand hygiene, and reduced patient transfers (Table 3 and Figure 1). The clinical consequences of these interventions ranged considerably, with daily cleaning preventing more than 16 times as many infections as the patient transfer intervention (25.9 vs 1.6). Screening at admission cost $1283 per QALY, while health care worker contact precautions and visitor hand hygiene interventions cost $123 264 and $5 730 987 per QALY, respectively. The visitor contact precautions intervention was dominated, with increased costs and decreased QALYs.

**Table 3.** Incremental Cost-effectiveness Ratios of Single and Bundled Intervention Strategies

Intervention strategy	Comparison	Mean incremental			Cost per HO-CDI averted, 2018 US $	Cost per QALY, 2018 US $
		Cost, 2018 US $	HO-CDI averted	QALY		
**Enhanced-level single interventions**						
Enhanced daily cleaning	Baseline	–358 268	25.9	36.8	Dominant	Dominant
Enhanced HCW CP	Baseline	87 080	0.4	0.7	217 266	123 264
Enhanced HCW HH	Baseline	–155 575	12.3	17.7	Dominant	Dominant
Enhanced patient HH	Baseline	–8235	4.2	6.3	Dominant	Dominant
Enhanced patient transfer	Baseline	–19 892	1.6	3.1	Dominant	Dominant
Enhanced screening	Baseline	23 763	13.4	18.5	1771	1283
Enhanced terminal cleaning	Baseline	–38 039	6.9	12.8	Dominant	Dominant
Enhanced visitor CP	Baseline	88 863	0.1	–0.2	982 995	Dominated
Enhanced visitor HH	Baseline	88 745	0.02	0.01	3 697 712	5 730 987
**Ideal-level single interventions**						
Ideal daily cleaning	Enhanced daily cleaning	38 707	1.6	2.1	24 071	18 399
Ideal HCW CP	Enhanced HCW CP	53 537	0.5	0.4	118 182	136 135
Ideal HCW HH	Enhanced HCW HH	–66 808	7.1	9.9	Dominant	Dominant
Ideal patient HH	Enhanced patient HH	–33 303	4.0	5.9	Dominant	Dominant
Ideal patient transfer	Enhanced patient transfer	7573	0.8	1.2	9772	6194
Ideal screening	Enhanced screening	56 150	0.4	0.6	158 080	100 084
Ideal terminal cleaning	Enhanced terminal cleaning	18 791	2.1	3.6	9093	5275
Ideal visitor CP	Enhanced visitor CP	55 896	–0.2	0.03	Dominated	1 669 089
Ideal visitor HH	Enhanced visitor HH	55 304	–0.1	–0.01	Dominated	Dominated
**Intervention bundles**						
HH bundle, ie, patient and HCW HH	Baseline	–188 164	15.3	22.0	Dominant	Dominant
HH bundle, ie, patient and HCW HH	HCW HH	–32 588	3.0	4.2	Dominant	Dominant
Environmental cleaning bundle, ie, daily and terminal cleaning	Baseline	–253 982	26.1	37.4	Dominant	Dominant
Environmental cleaning bundle, ie, daily and terminal cleaning	Daily cleaning	104 285	0.2	0.6	494 712	170 469
Patient-centered bundle, ie, screening, patient HH, patient transfer	Baseline	–35 594	19.9	28.3	Dominant	Dominant
Daily cleaning, screening	Baseline	–172 979	30.9	43.0	Dominant	Dominant
Daily cleaning, screening	Daily cleaning	185 288	5.0	6.3	36 769	29 616
Daily cleaning, screening, HCW HH	Daily cleaning, screening bundle	79 998	1.1	1.6	74 293	50 196
Daily cleaning, screening, HCW HH, patient HH	Daily cleaning, screening, HCW HH bundle	56 836	0.3	0.4	214 315	146 792
Daily cleaning, screening, HCW HH, patient HH, terminal cleaning	Daily cleaning, screening, HCW HH, patient HH bundle	134 921	0.03	0.2	4 164 243	758 618
Daily cleaning, screening, HCW HH, patient HH, terminal cleaning, patient transfer	Daily cleaning, screening, HCW HH, patient HH, terminal cleaning bundle	17 761	0.04	0.1	422 885	221 009

Abbreviations: CP, contact precautions; HCW, health care worker; HH, hand hygiene; HO-CDI, hospital-onset *Clostridioides difficile* infection; QALY, quality-adjusted life year.

Improving from enhanced to ideal intervention levels offered only small clinical benefits for most interventions (Table 3). It was cost saving and most effective for ideal health care worker and patient hand hygiene, averting an additional 7.1 and 4.0 HO-CDIs a year, respectively, compared with enhanced interventions. The ideal level was cost-effective for daily cleaning ($18 399/QALY), terminal cleaning ($5275/QALY), and patient transfer ($6194/QALY) at a willingness-to-pay threshold of $50 000/QALY.

Cost-effectiveness of the bundle strategies varied based on a bundle’s intervention components (Table 3). Adding patient hand hygiene to the health care worker hand hygiene intervention was cost saving, saving a mean of $32 588 and 4.2 QALYs annually in the model 200-bed hospital compared with the health care worker hand hygiene intervention alone. When screening, health care worker hand hygiene, and patient hand hygiene interventions were sequentially added to daily cleaning to form 2-, 3-, and 4-pronged bundles, the ICERs for these additions were $29 616, $50 196, and $146 792 per QALY, respectively.

**Figure 2.**
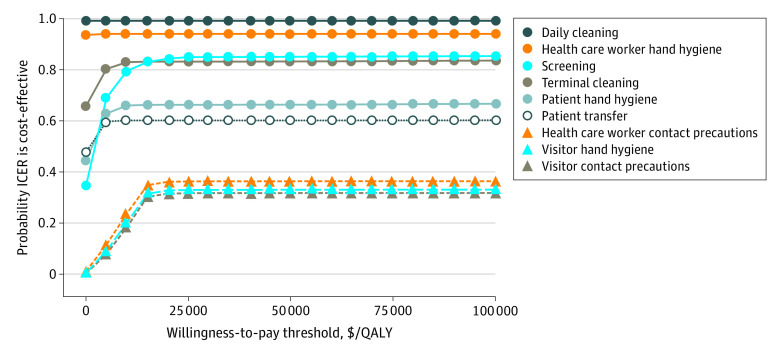
Acceptability Curve Based on 5000 Runs of Each Intervention at 21 Willingness-to-Pay Thresholds ICER indicates incremental cost-effectiveness ratio; and QALY, quality-adjusted life-year.

We also evaluated the percentage of times each intervention was cost-effective at 21 willingness-to-pay thresholds. These results are presented as an acceptability curve (Figure 2). Daily cleaning consistently had the greatest proportion of runs that were cost-effective, with 99% of runs cost-effective at a willingness-to-pay threshold of $5000 per QALY.

Detailed results of the 1-way sensitivity analyses and probabilistic sensitivity analysis are included in eFigure 2, eFigure 3, eFigure 4, and eTable 3 in the Supplement. The trends in comparative cost-effectiveness were stable across most variations in cost and utility parameters. The 5 cost-saving interventions were most sensitive to hospitalization costs (eFigure 2 in the Supplement). Screening at admission was most sensitive to increased costs of polymerase chain reaction testing. Visitor hand hygiene and health care worker contact precautions were most sensitive to changes in age-related utility values (eFigure 3 in the Supplement). Most notably, in the probabilistic sensitivity analysis (eFigure 4 in the Supplement), the patient-centered intervention bundle (comprised of screening at admission, patient hand hygiene, and patient transfer) changed from cost-saving to a cost of $245/QALY, and the visitor hand hygiene intervention became dominated (compared with $5 730 987/QALY) (eTable 3 in the Supplement).

## Discussion

In this model-based economic evaluation, daily cleaning, health care worker hand hygiene, patient hand hygiene, terminal cleaning, and reduced patient transfers were all found to be cost saving. Daily cleaning was the most clinically effective and cost-effective intervention by far, saving $358 268, 25.9 infections, and 36.8 QALYs annually in the 200-bed model hospital. In comparison with the other existing *C difficile* simulation models, Brain et al^9^ found that a cleaning and hand hygiene bundle had the greatest increase in QALYs and was the most cost-saving of 9 bundle strategies. Nelson et al^8^ reported that increasing environmental cleaning within the context of multi-intervention bundles resulted in minimal gains in effectiveness. However, their bundle strategies included up to 6 interventions simultaneously and are not comparable with an isolated daily cleaning intervention. Similarly, a recent multicenter trial by Ray et al^145^ found that reduction of *C difficile* environmental cultures did not correlate with reduced infection rates. However, this study is also not comparable, given that it targeted sporicidal daily cleaning only in known CDI rooms and did not change practices for non-CDI patient rooms and hospital common rooms. Thus, it appears that blocking asymptomatic transmission by using sporicidal products hospitalwide may be essential to obtaining a reduction in HO-CDI rates.

Among all the interventions we modeled, health care worker hand hygiene is the most well studied and has been shown to be cost saving in several prior contexts. Chen et al^146^ reported that every dollar spent on their hospital’s 4-year hand hygiene program resulted in a $32.73 return on investment (2018 USD). Likewise, Pittet et al^147^ found that hand hygiene needed to account for less than 1% of the concurrent decline in hospital-associated infections at their institution to be cost saving. Our results are also in line with the prior modeling studies. Nelson et al^8^ reported that adding health care worker hand hygiene to existing bundles increased total QALYs with few additional costs, and health care worker hand hygiene was a key component of the most cost-saving cleaning and hygiene bundle in the study by Brain et al.^9^

*C difficile* screening has also recently been shown to be highly effective at reducing HO-CDI in real-world and modeling contexts.^6,10,11,148,149^ This intervention was highly cost-effective in our model, at a cost of $1283/QALY and is similar to the results of the study by Bartsch et al,^124^ in which screening cost less than $310/QALY (2018 USD).^124^ Both are likely conservative estimates because the cost-effectiveness of screening is expected to increase if the intervention is targeted to high-risk populations. In fact, when Saab et al^149^ modeled a *C difficile* screening and treatment intervention exclusively for patients with cirrhosis, costs were found to be 3.54 times lower than under baseline conditions.

The Veterans Affairs methicillin-resistant *Staphylococcus aureus* (MRSA) screening bundle, instituted at Veterans Affairs hospitals nationwide in 2007, provides a precedent for large-scale screening implementation. It ultimately had a 96% participation rate and reduced MRSA by 45% among patients not in the intensive care unit patients and 62% among patients in the intensive care unit.^90^ The cost-effectiveness of this intervention was calculated at between $31 979 and $64 926 per life-year saved (2018 USD).^97^ Given the evidence from our study and others,^124,149^ we expect that screening for *C difficile* would be even more cost-effective than the Veteran Affairs MRSA initiative. However, additional work is needed to identify which populations to target before widespread implementation.

While screening is not yet standard practice, contact precautions are a mainstay of *C difficile* infection prevention programs.^3^ They are recommended by the Society for Healthcare Epidemiology of America for both health care workers and visitors of patients with CDI.^150,151^ However, evidence for these guidelines is based primarily on studies of other pathogens and theoretical transmission concerns,^108,152^ given that *C difficile*–targeted studies are lacking. In our study, we found neither health care worker nor visitor contact precautions to be cost-effective. The enhanced-level health care worker contact precautions intervention cost $123 264 per QALY, with another $136 135 per QALY for the ideal-level implementation. The results were even worse for visitor contact precaution interventions, with the enhanced level being dominated and the ideal level costing $1 669 089 per QALY. Thus, it is likely that the screening intervention, which, as modeled, prompts the use of visitor and health care worker contact precautions for asymptomatic colonized patients, would be even more cost-effective if contact precautions were not used for asymptomatic patients who test positive.

Recognizing that all hospitals operate in an environment of constrained resources, support must be shifted from minimally effective, high-cost interventions, such as visitor contact precautions, to more innovative, cost-effective solutions. For example, patient hand hygiene, which is rarely incorporated into *C difficile* bundles,^3^ was 1 of only 2 interventions to be cost saving at both the enhanced and ideal level. It was also cost saving compared with health care worker hand hygiene alone. In fact, all 2-pronged intervention bundles investigated in this study were cost saving. However, incremental intervention cost-effectiveness decreased beyond 2-intervention bundles. Adding subsequent interventions to the 2-pronged daily cleaning and screening at admission bundle came at an ICER of $50 196/QALY for the third strategy, $146 792/QALY for the fourth strategy, and $758 618/QALY for the fifth strategy.

The recommendation to implement a smaller number of highly effective interventions runs contrary to the current infection control climate. A recent review of CDI bundles found that more than half of bundles include 6 or more components, with a minimum of 3 and maximum of 8 interventions.^3^ Given the lack of evidence and guidelines surrounding bundle composition, it is not surprising that institutions seek to maximize CDI reduction by implementing increasingly larger bundled strategies. However, our results provide evidence that continuing to increase bundles without accounting for the cost and effectiveness of individual components may be counterproductive, depending on institutional priorities and cost constraints. Instead, institutions should consider streamlining their infection control initiatives and may opt to focus on a smaller number of highly cost-effective interventions.

It is important to note that while many of the interventions in this study were cost saving, they are not without upfront costs. Even at the enhanced level, each intervention required the employment of additional infection control nursing staff. These individuals have the critical responsibility of coordinating implementation, assessing compliance, providing direct frontline feedback, and iteratively evaluating intervention effectiveness. Hospital administrative buy-in and financial support is key to both the initial implementation of an intervention and sustaining its long-term success.

### Limitations

This study has limitations. The cost-effectiveness results presented in this study are inherently dependent on the quality of our agent-based model, which underwent rigorous verification and validation processes.^6^ It suffers from limitations of the original model, such as assuming transmission of a generic *C difficile* strain and the lack of an antibiotic stewardship intervention. Particularly relevant to this study, we did not stratify CDI by severity or include complications such as colitis or toxic megacolon. By evaluating all cases using a utility value that corresponds to mild to moderate CDI, we likely underestimate the true cost-effectiveness of these interventions.

## Conclusions

To our knowledge, this was the first *C difficile* cost-effectiveness analysis to compare standard infection control strategies and emerging patient-centered interventions. In a field that lacks specific guidance regarding the cost-effectiveness of interventions targeting *C difficile*, this study provides critical evidence regarding where to allocate limited resources for the greatest potential success. Daily sporicidal cleaning is among several promising, cost-saving strategies that should be prioritized over minimally effective, costly strategies, such as visitor contact precautions. Maintaining the status quo, focused on large, multipronged bundles with variable efficacy, will continue to shift limited resources away from more productive, cost-saving strategies that have greater potential to improve patient outcomes.

## Notes

Supplement. **eAppendix. **Agent-Based Simulation Model **eTable 1. **Transmission Parameters Estimates **eTable 2. **Discounted QALY Analysis **eTable 3. **Probabilistic Sensitivity Analysis Results of Incremental Cost-effectiveness Ratios for 100 000 Runs **eFigure 1. **Schematic of the 200-Bed Model Hospital and Possible Agent Movement **eFigure 2. **One-Way Sensitivity Analysis of Cost-Saving Interventions **eFigure 3. **One-Way Sensitivity Analysis of Cost-effective Interventions **eFigure 4. **Incremental Cost-effectiveness of 100 000 Runs of the Probabilistic Sensitivity Analysis **eReferences. **
