# Supplementary material for: Evaluation of the Cost-effectiveness of Infection Control Strategies to Reduce Hospital-Onset Clostridioides difficile Infection
Source: JAMA Netw Open. 2020 Aug 13;3(8):e2012522. doi: 10.1001/jamanetworkopen.2020.12522 (PMC7426752; doi:10.1001/jamanetworkopen.2020.12522)
Supplement: Supplement. — eAppendix. Agent-Based Simulation Model eTable 1. Transmission Parameters Estimates eTable 2. Discounted QALY Analysis eTable 3. Probabilistic Sensitivity Analysis Results of Incremental Cost-effectiveness Ratios for 100 000 Runs eFigure 1. Schematic of the 200-Bed Model Hospital and Possible Agent Movement eFigure 2. One-Way Sensitivity Analysis of Cost-Saving Interventions eFigure 3. One-Way Sensitivity Analysis of Cost-effective Interventions eFigure 4. Incremental Cost-effectiveness of 100 000 Runs of the Probabilistic Sensitivity Analysis eReferences. [file jamanetwopen-3-e2012522-s001.pdf]

Supplementary Online Content

Barker AK, Scaria E, Safdar N, Alagoz O. Evaluation of the cost-effectiveness of infection control to reduce hospital-onset *Clostridioides difficile* infection. *JAMA Netw Open*. 2020;3(8):e2012522 doi:10.1001/jamanetworkopen.2020.12522

eAppendix. Agent-Based Simulation Model
eTable 1. Transmission Parameters Estimates
eTable 2. Discounted QALY Analysis
eTable 3. Probabilistic Sensitivity Analysis Results of Incremental Cost-effectiveness Ratios for 100 000 Runs
eFigure 1. Schematic of the 200-Bed Model Hospital and Possible Agent Movement
eFigure 2. One-Way Sensitivity Analysis of Cost-Saving Interventions
eFigure 3. One-Way Sensitivity Analysis of Cost-effective Interventions
eFigure 4. Incremental Cost-effectiveness of 100 000 Runs of the Probabilistic Sensitivity Analysis
eReferences.

This supplementary material has been provided by the authors to give readers additional information about their work.

© 2020 Barker AK et al. *JAMA Network Open.*

eAppendix. Agent-Based Simulation Model

Our agent-based model simulates *C. difficile* transmission in a 200-bed adult inpatient hospital. Full model details including parameter estimation, calibration, verification, and validation are provided in a prior publication, from which this is adapted.[2] The model represents a general US hospital, however it could be adapted to represent any specific real-world institution. To maximize generalizability, model input and intervention parameters were derived from over 100 peer-reviewed studies, published through April 2017 (selected parameters included in Table 1). The modeled hospital is comprised of 10 identical wards (eFigure 1), each of which contains 20 single-bed patient rooms, a nursing station, physician workroom, and common room for visitors and patients. Each model run includes a 28-day warmup period, followed by one simulated year of data collection. Among other measures, the model outputs the annual number of hospital-onset *C. difficile* infections (HO-CDI), defined as symptomatic diarrhea plus a positive laboratory test result on a specimen collected more than three days after hospital admission.[3] The cost per HO-CDI averted and cost per quality-adjusted life year (QALY) saved are the two primary outcomes of the cost-effectiveness study. The model has four different types of agents as described below.

Patients: Upon arrival to the hospital, patients are assigned a specific ward and room number. Each patient is also assigned an initial clinical state, which is subsequently updated every 6 hours based on probabilities in the model's discrete-time Markov chain. This discrete-time Markov chain was selected after extensive calibration from a subset of ten transition matrices run on the model simultaneously to account for population heterogeneity, as described by Barker, et al.[2] and Codella, et al.[4] The nine possible clinical states for patient agents include five in which the patient is not contagious: susceptible to colonization, non-susceptible to colonization, exposed to *C. difficile*, cleared (includes recent clearing of prior infection or colonization), and death, as well as four in which they are contagious: colonized (asymptomatic), infected (symptomatic), recolonized (sustained colonization, but asymptomatic), and recurrent infection (return of symptoms). Contagious patients can transmit *C. difficile* to nurses, doctors, visitors, and any environment they come into contact with, as well as other patients they meet in the ward common room. Conversely, they can become exposed from contaminated nurses, doctors, other patients, and the environment. Only one patient is assigned to each single-bed room at a time. Patients may change rooms within or between wards while at the hospital. These intra- and inter-ward transfers occur based on the patient transfer probabilities listed in Table 1.

Visitors: All visitors are assigned to a single patient and stay with them for an average visit duration of 15 minutes.[5–8] Visits occur in their patient's room or the ward common room. All visitors exit through the ward common room to leave the hospital. Visitors can become contaminated by the patient or environment they visit and can subsequently expose the environment in the patient's room or ward common room.

Nurses: Four nurses are assigned to each ward.[5,9–11] They are based at their ward nursing workstation and leave it to care for patients in the patient rooms. Nurses only visit patients on their home ward. They spend an average of 4.7 minutes at each patient encounter.[5,12–14] Patients are visited by nurses an average of five times every six hours.[5,12,15,16] Like visitors, nurses can become contaminated by the patient or environment they visit. Once contaminated, they can subsequently expose other patients or the environment in patient rooms or the nursing workstation.

Doctors: Two doctors are assigned to each ward.[5,17] They are based at their home ward physician workroom, but leave it to care for patients throughout the hospital. They spend an average of 10.8 minutes at each patient encounter.[5,12–14] Each patient is visited by a doctor an average of one time every six hours.[5,12,15,16] Like visitors and nurses, doctors can become contaminated by the patient or environment they visit. Once contaminated, they can subsequently expose other patients or the environment in patient rooms or the physician workroom.

***C. difficile* transmission:** Transmission occurs due to transient exposure to *C. difficile* spores from contagious patients and contaminated visitors, nurses, doctors, and the environment. The probability of contamination is proportional to the duration of exposure. Infection control interventions decrease *C. difficile* transmission by preventing (example: contact precautions) or clearing (example: hand hygiene, environmental cleaning) exposure and contamination.

Infection control interventions: Nine single interventions and eight multi-intervention bundles were modeled (selected parameters included in Table 1). Each single intervention was modeled at baseline, enhanced, and ideal implementation levels.

1. Healthcare worker hand hygiene improved nurse and physician hand hygiene compliance and increased the utilization of soap and water (versus alcohol-based hand rub) for known *C. difficile* patients.

2 Patient hand hygiene had similar effects on compliance and soap and water usage as the healthcare worker hand hygiene intervention, but for patient practices.

3. Visitor hand hygiene had similar effects on compliance and soap and water usage as the healthcare worker and patient hand hygiene interventions, but for visitor practices.

4. Healthcare worker contact precautions extended contact precautions for *C. difficile* patients until patient discharge, improved nurse and physician compliance, and increased the effectiveness of precautions at preventing contamination via an educational component aimed at improving donning and doffing techniques.

5. Visitor contact precautions had similar effects on contact precaution duration, compliance, and effectiveness as the healthcare worker contact precaution intervention, only for visitor practices.

6. Screening involved evaluating all asymptomatic patients for *C. difficile* colonization within 24 hours of admission via polymerase chain reaction testing of a stool sample or rectal swab. All *C. difficile* infection control policies except treatment were then implemented for any patients identified as colonized.

7. Daily cleaning increased the proportion of rooms cleaned daily by environmental cleaning staff and utilized sporicidal product in all patient, staff, and common rooms hospital-wide, regardless of *C. difficile* status.

8. Terminal cleaning increased the proportion of rooms cleaned by environmental cleaning staff at discharge, intra-ward, or inter-ward room transfer and utilized sporicidal product in all patient rooms hospital-wide, regardless of *C. difficile* status.

9. Reducing patient transfers decreased the rate of intra- and inter-ward transfers and prohibited room transfers for known *C. difficile* colonized or infected patients. This decreased the terminal cleaning burden for environmental cleaning staff and thus indirectly increased terminal cleaning rates.

Multiple intervention bundle strategies were developed in a stepwise approach, sequentially adding the most clinically effective enhanced single interventions until no additional effectiveness was obtained by enlarging the bundle. Hand hygiene (healthcare worker and patient hand hygiene), environmental cleaning (daily and terminal cleaning), and patient-centered (screening, patient hand hygiene, and patient transfer) bundles were also modeled, based on expert opinion that these interventions were likely to be implemented simultaneously in bundle form.

Fixed cost: Enhanced patient transfer had the lowest staffing requirement, at 0.25 full-time equivalents (FTE). Enhanced daily cleaning, terminal cleaning, and screening, and ideal patient transfer required 0.5. FTE. Enhanced healthcare worker, patient, and visitor hand hygiene, enhanced healthcare worker and visitor contact precautions, and ideal daily and terminal cleaning, required 0.75 FTE. Ideal screening required 1 FTE. Ideal healthcare worker, patient, and visitor hand hygiene and ideal healthcare worker and visitor contact precautions required 1.25 FTE. These staffing requirements reflect a minimum 0.5 FTE for all ideal level interventions and an additional 0.25 FTE for interventions requiring a serial implementation campaign. This additional 0.25 FTE was not included when interventions were bundled, to account for synergy between campaigns. Otherwise, all fixed costs were added at face value, regardless of the number of interventions bundled.

Model verification and validation: Model verification included a structured walkthrough of the code by members of the research team and iterative dynamic testing throughout the coding process using traces and animation. The validation process included parameter sensitivity analyses, cross-validation of results with the existing *C. difficile* infection control literature, face validation of the model's logic, transmission dynamics, parameters, and results by content experts during and after model development, and calibration of the discrete-time Markov chain.

© 2020 Barker AK et al. *JAMA Network Open.*

eTable 1. Transmission Parameters Estimates

Parameter Estimates	Mean; Triangular distribution (min, max)	Source
Probability during an encounter that patient has physical contact with	Another patient: 5% per 30 min; (1, 15)<br>Nurse: 36% per 4.7 min (26, 46)<br>Doctor: 69% per 10.8 min (59,79)<br>Visitor: 65% per 15 min (55,75)	Patient:patient; EO<br>Others: [5]
Probability during an encounter that environment has physical contact with	Patient: 100%; constant; --<br>Nurse: 70% per 4.7 min; (60, 80%)<br>Doctor: 90% per 10.8 min; (80, 100%)<br>Visitor: 93% per 15 min; (83, 100%)	[5]
C. difficile transfer efficiency	Person:person 30%; (15, 45%)<br>Person:environment 44%; (29, 59%)	Person:person: [18]<br>Person:environment: [19]
Probability that skin is contaminated on	Colonized patient: 38%; (15, 60%)<br>Active CDI patient: 70%; (60, 80%)	[20–22]
Probability that patient room is contaminated for	Colonized patient: 19%; (14, 35%)<br>Active CDI patient: 47%; (36, 60%)<br>Non-*C. difficile* patient: 7%; (5, 15%)	Colonized: [22–24]<br>Active CDI: [22-27]<br>Non-*C. difficile:* [23,26,27]

C. difficile, Clostridioides difficile; EO, expert opinion; adapted from Barker, et al.[2]

© 2020 Barker AK et al. *JAMA Network Open.*

eTable 2. Discounted QALY Analysis

Intervention Strategy	Strategy compared to	Mean incremental cost (2018 USD)	Mean incremental QALY	Cost per QALY (2018 USD)
Enhanced-level single interventions				
Enhanced daily cleaning	Baseline	-$358,268	25.3	Dominant
Enhanced HCW CP	Baseline	$87,080	0.5	$179,019
Enhanced HCW HH	Baseline	-$155,575	12.2	Dominant
Enhanced patient HH	Baseline	-$8,235	4.4	Dominant
Enhanced patient transfer	Baseline	-$19,892	2.2	Dominant
Enhanced screening	Baseline	$23,763	12.8	$1,864
Enhanced terminal cleaning	Baseline	-$38,039	8.8	Dominant
Enhanced visitor CP	Baseline	$88,863	-0.1	Dominated
Enhanced visitor HH	Baseline	$88,745	0.01	$8,239,801
Ideal level single interventions				
Ideal daily cleaning	Enhanced daily cleaning	$38,707	1.4	$26,723
Ideal HCW CP	Enhanced HCW CP	$53,537	0.3	$197,789
Ideal HCW HH	Enhanced HCW HH	-$66,808	6.8	Dominant
Ideal patient HH	Enhanced patient HH	-$33,303	4.0	Dominant
Ideal patient transfer	Enhanced patient transfer	$7,573	0.8	$8,997
Ideal screening	Enhanced screening	$56,150	0.4	$145,445
Ideal terminal cleaning	Enhanced terminal cleaning	$18,791	2.5	$7,663
Ideal visitor CP	Enhanced visitor CP	$55,896	0.02	$2,429,054
Ideal visitor HH	Enhanced visitor HH	$55,304	-0.006	Dominated
Bundled interventions				
Patient and HCW HH (hand hygiene bundle)	Baseline	-$188,164	15.1	Dominant
Patient and HCW HH (hand hygiene bundle)	HCW HH	-$32,588	2.9	Dominant
Daily and terminal cleaning (cleaning bundle)	Baseline	-$253,982	25.7	Dominant
Daily and terminal cleaning (cleaning bundle)	Daily cleaning	$104,285	0.4	$247,668
Screening, patient HH, patient transfer (patient-centered bundle)	Baseline	-$35,594	19.5	Dominant
Daily cleaning, screening	Baseline	-$172,979	29.6	Dominant
Daily cleaning, screening	Daily cleaning	$185,288	4.3	$43,021
Daily cleaning, screening, HCW HH	Daily cleaning, screening bundle	$79,998	1.1	$72,919
Daily cleaning, screening, HCW HH, patient HH	Daily cleaning, screening, HCW HH bundle	$56,836	0.3	$213,110
Daily cleaning, screening, HCW HH, patient HH, terminal cleaning	Daily cleaning, screening, HCW HH, p atient HH bundle	$134,921	0.1	$1,103, 008
Daily cleaning, screening, HCW HH, patient HH, terminal cleaning, patient transfer	Daily cleaning, screening, HCW HH, p atient HH, terminal cleaning bundle	$17,761	0.1	$321,654

The trends regarding intervention cost effectiveness remain the same, while the cost per QALY increases for any given intervention under the discounted QALY analysis. CP, Contact precautions; HCW, Healthcare worker; HH, Hand hygiene; QALY, Quality-adjusted life year; USD, United States dollar

© 2020 Barker AK et al. *JAMA Network Open.*

eTable 3. Probabilistic Sensitivity Analysis Results of Incremental Cost-effectiveness Ratios for 100 000 Runs

Intervention Strategy	Strategy compared to	Mean incremental cost (2018 USD)	Mean incremental HO-CDI averted	Mean incremental QALY	Cost per HO-CDI averted (2018 USD)	Cost per QALY (2018 USD)
Enhanced-level single interventions						
Enhanced daily cleaning	Baseline	-$356,261	25.8	37.2	Dominant	Dominant
Enhanced HCW CP	Baseline	$87,063	0.5	0.7	$183,344	$125,813
Enhanced HCW HH	Baseline	-$156,376	12.4	18.0	Dominant	Dominant
Enhanced patient HH	Baseline	-$8,055	4.2	6.4	Dominant	Dominant
Enhanced patient transfer	Baseline	-$20,449	1.7	3.2	Dominant	Dominant
Enhanced screening	Baseline	$65,661	13.5	18.8	$4,874	$3,499
Enhanced terminal cleaning	Baseline	-$38,335	7.0	13.0	Dominant	Dominant
Enhanced visitor CP	Baseline	$88,458	0.008	-0.02	$11,563,202	Dominated
Enhanced visitor HH	Baseline	$88,212	0.01	-0.07	$6,041,920	Dominated
Ideal level single interventions						
Ideal daily cleaning	Enhanced daily cleaning	$38,805	1.6	2.3	$24,879	$17,100
Ideal HCW CP	Enhanced HCW CP	$53,504	0.3	0.4	$162,870	$126,965
Ideal HCW HH	Enhanced HCW HH	-$64,536	7.0	9.9	Dominant	Dominant
Ideal patient HH	Enhanced patient HH	-$34,115	4.0	6.0	Dominant	Dominant
Ideal patient transfer	Enhanced patient transfer	$7,957	0.7	1.3	$11,219	$6,009
Ideal screening	Enhanced screening	$56,789	0.4	0.4	$157,930	$127,822
Ideal terminal cleaning	Enhanced terminal cleaning	$18,116	2.0	3.4	$9,258	$5,256
Ideal visitor CP	Enhanced visitor CP	$55,877	0.0005	-0.03	$103,476,304	Dominated
Ideal visitor HH	Enhanced visitor HH	$55,919	-0.001	-0.009	Dominated	Dominated
Bundled interventions						
Patient and HCW HH (HH bundle)	Baseline	-$187,371	15.3	22.3	Dominant	Dominant
Patient and HCW HH (HH bundle)	HCW HH	-$30,996	2.9	4.3	Dominant	Dominant
Daily and terminal cleaning (environmental cleaning bundle)	Baseline	-$252,055	26.0	37.8	Dominant	Dominant
Daily and terminal cleaning environmental cleaning bundle)	Daily cleaning	$104,207	0.2	0.6	$555,118	$188,412
Screening, patient HH, patient transfer (patient-centered bundle)	Baseline	$6,979	19.9	28.5	$351	$245
Daily cleaning, screening	Baseline	-$129,150	30.8	43.6	Dominant	Dominant
Daily cleaning, screening	Daily cleaning	$227,112	5.0	6.4	$45,586	$35,430
Daily cleaning, screening, HCW HH	Daily cleaning, screening bundle	$80,959	1.1	1.5	$75,204	$53,141
Daily cleaning, screening, HCW HH, patient HH	Daily cleaning, screening, HCW HH bundle	$56,803	0.3	0.5	$180,579	$111,279
Daily cleaning, screening, HCW HH, patient HH, terminal cleaning	Daily cleaning, screening, HCW HH, patient HH bundle	$134,946	0.03	0.1	$4,069,547	$2,253,251
Daily cleaning, screening, HCW HH, patient HH, terminal cleaning, patient transfer	Daily cleaning, screening, HCW HH, patient HH, terminal clea ning bundle	$17,388	0.0002	0.05	$115,921,899	$349,898

CP, Contact precautions; HCW, Healthcare worker; HH, Hand hygiene; QALY, Quality-adjusted life year; USD, United States dollar

© 2020 Barker AK et al. *JAMA Network Open.*

eFigure 1. Schematic of the 200-Bed Model Hospital and Possible Agent Movement

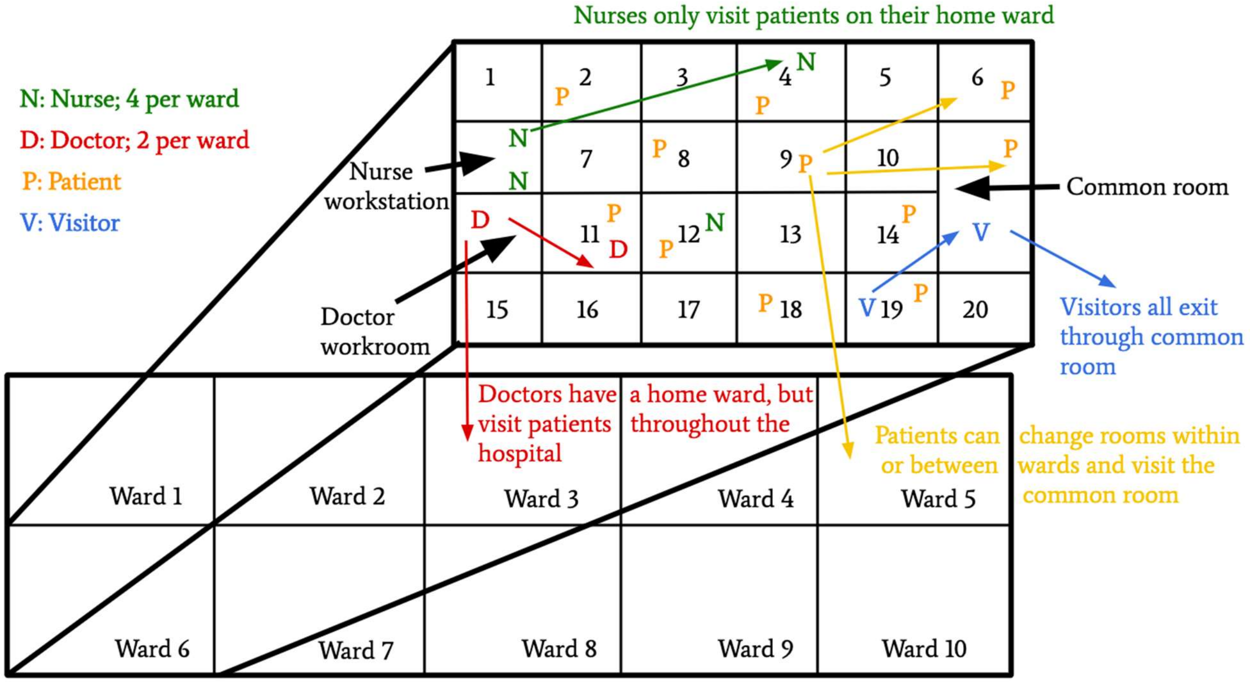

© 2020 Barker AK et al. *JAMA Network Open.*

eFigure 2. One-Way Sensitivity Analysis of Cost-Saving Interventions

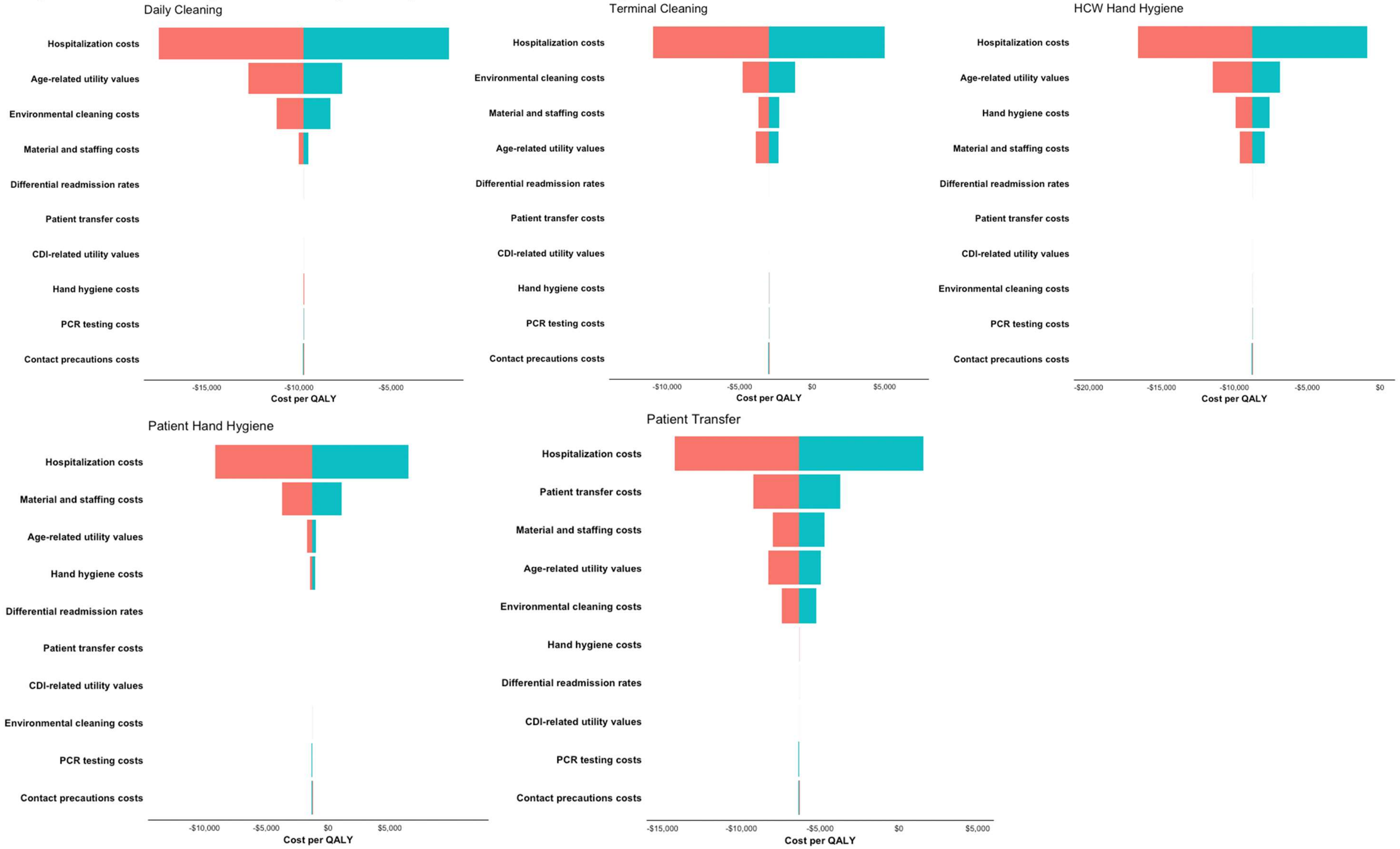

One-way sensitivity analyses were based on 5,000 runs of each enhanced level cost-saving intervention, compared to baseline. The interventions were most sensitive to hospitalization costs. CDI, *Clostridioides difficile* infection; HCW: Healthcare worker; PCR, polymerase chain reaction; QALY, Quality-adjusted life year.

© 2020 Barker AK et al. *JAMA Network Open.*

eFigure 3. One-Way Sensitivity Analysis of Cost-effective Interventions

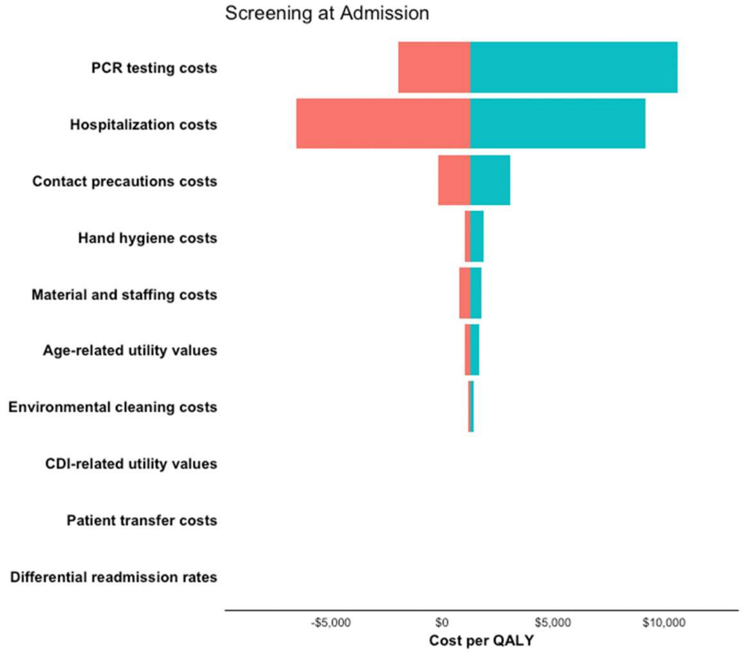

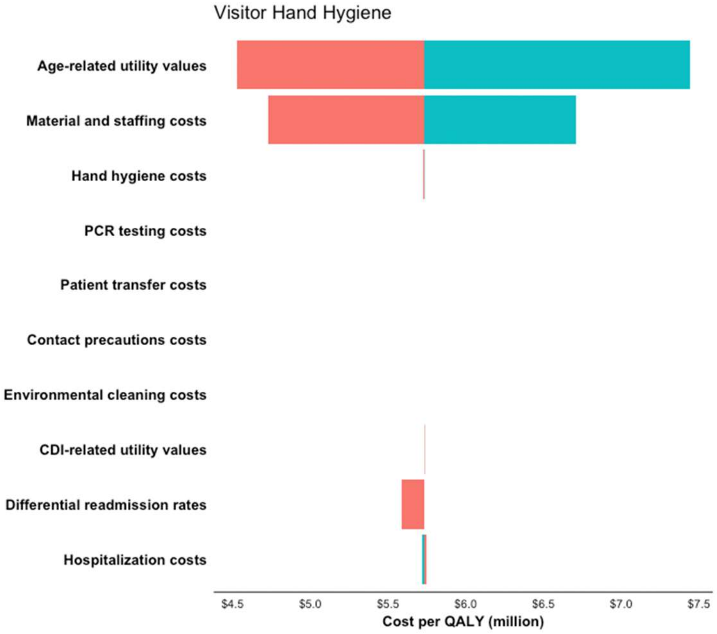

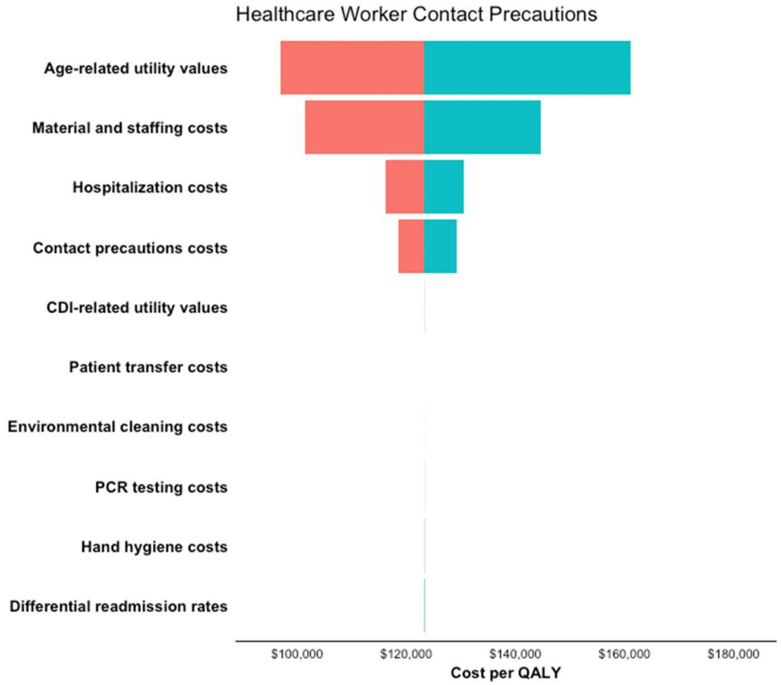

One-way sensitivity analyses were based on 5,000 runs of both enhanced level interventions, compared to baseline. Screening at admission was most sensitive to increased PCR testing costs. The visitor hand hygiene and healthcare worker contact precautions interventions resulted in minimal improvement in QALYs saved, thus were most sensitive to changes in age-related utility values. Visitor contact precautions is not shown, because the intervention is dominated. CDI, *Clostridioides difficile* infection; PCR, polymerase chain reaction; QALY, Quality-adjusted life year

© 2020 Barker AK et al. *JAMA Network Open.*

eFigure 4. Incremental Cost-effectiveness of 100 000 Runs of the Probabilistic Sensitivity Analysis

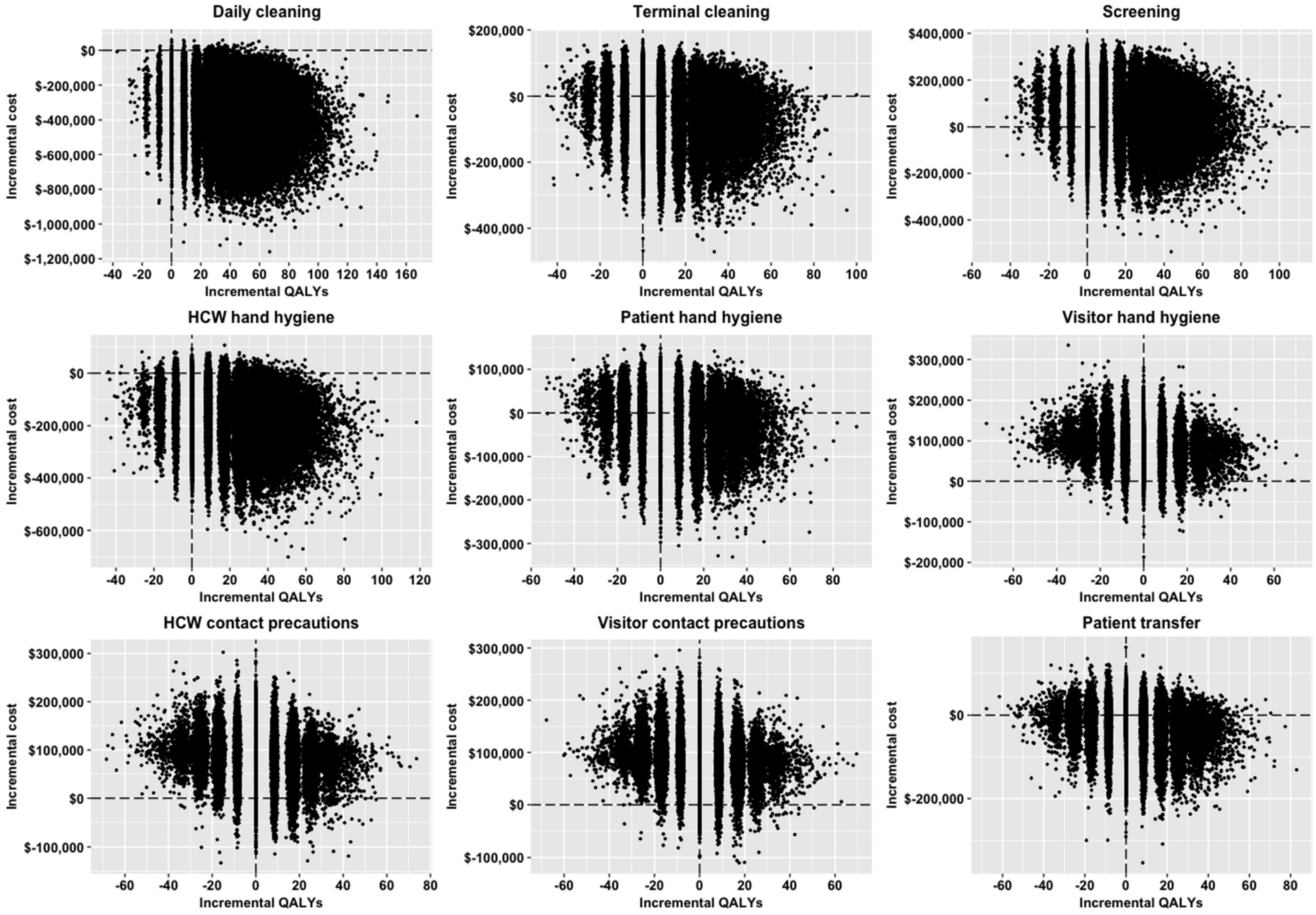

HCW, Healthcare worker; QALY, Quality-adjusted life year

© 2020 Barker AK et al. *JAMA Network Open.*

eReferences.

1. Husereau D, Drummond M, Petrou S, et al. Consolidated Health Economic Evaluation Reporting Standards (CHEERS) Statement. *Value Health*. 2013;16(2):e1-e5.
2. Barker AK, Alagoz O, Safdar N. Interventions to reduce the incidence of hospital-onset Clostridium difficile infection: An agent-based modeling approach to evaluate clinical effectiveness in adult acute care hospitals. *Clin Infect Dis*. 2018;66(8):1192-1203.
3. Centers for Disease Control and Prevention. Multidrug-Resistant Organism & Clostridioides difficile Infection (MDRO/CDI) Module. https://www.cdc.gov/nhsn/PDFs/pscManual/12pscMDRO_CDADcurrent.pdf. Accessed April 10, 2019.
4. Codella J, Safdar N, Heffernan R, Alagoz O. An agent-based simulation model for Clostridium difficile infection control. *Med Decis Mak.* 2015;35(2):211-229.
5. Cohen B, Hyman S, Rosenberg L, Larson E. Frequency of Patient Contact with Health Care Personnel and Visitors: Implications for Infection Prevention. *Jt Comm J Qual Patient Saf.* 2012;38(12):560-565.
6. Eriksson T, Bergbom I. Visits to intensive care unit patients--frequency, duration and impact on outcome. *Nurs Crit Care*. 2007;12(1):20-26.
7. Gonzalez CE, Carroll DL, Elliott JS, Fitzgerald PA, Vallent HJ. Visiting preferences of patients in the intensive care unit and in a complex care medical unit. *Am J Crit Care.*2004;13(3):194-198.
8. Fumagalli S, Boncinelli L, Lo Nostro A, et al. Reduced cardiocirculatory complications with unrestrictive visiting policy in an intensive care unit: results from a pilot, randomized trial. *Circulation*. 2006;113(7):946-952.
9. California Code of Regulations. Nurse service staff 2003. 22 C.C.R., Sec. 70217. July 2003. https://govt.westlaw.com/calregs/Document/I8612C410941F11E29091E6B951DDF6CE?viewType=FullText&originationContext=documenttoc&transitionType=CategoryPageItem&contextData=(sc.Default&bhcp=1. Accessed May 10, 2019.
10. Spetz J, Donaldson N, Aydin C, Brown DS. How Many Nurses per Patient? Measurements of Nurse Staffing in Health Services Research. *Health Serv Res*. 2008;43(5 Pt 1):1674-1692.
11. Aiken LH, Clarke SP, Sloane DM, Sochalski J, Silber JH. Hospital nurse staffing and patient mortality, nurse burnout, and job dissatisfaction. *JAMA*. 2002;288(16):1987-1993.
12. Morgan DJ, Pineles L, Shardell M, et al. The effect of contact precautions on healthcare worker activity in acute care hospitals. *Infect Control Hosp Epidemiol*. 2013;34(1):69-73.
13. Evans HL, Shaffer MM, Hughes MG, et al. Contact isolation in surgical patients: A barrier to care? *Surgery*. 2003;134(2):180-188.
14. Barker AK, Codella J, Ewers T, Dundon A, Alagoz O, Safdar N. Changes to physician and nurse time burdens when caring for patients under contact precautions. *Am J Infect Control*. 2017;45(5):542-543.
15. Rubin MA, Jones M, Leecaster M, et al. A Simulation-Based Assessment of Strategies to Control Clostridium Difficile Transmission and Infection. *PLoS ONE*. 2013;8(11).
16. McArdle FI, Lee RJ, Gibb AP, Walsh TS. How much time is needed for hand hygiene in intensive care? A prospective trained observer study of rates of contact between healthcare workers and intensive care patients. *J Hosp Infect*. 2006;62(3):304-310.
17. American Hospital Association. AHA Hospital Statistics, 2016. 2016 Chicago, IL: American Hospital Association.
18. Jabber U, Leischner J, Kasper D, et al. Effectiveness of alcohol-based hand rubs for removal of *Clotridum difficile* spores from hands. *Infect Control Hosp Epidemiol.* 2010;31:565-570.
19. Lopez GU, Gerba CP, Tamini AH, Kitajima M, Maxwell SL, Rose JB. Transfer efficiency of bacteria and viruses from porous and nonporous fomites to fingers under different relative humidity conditions. *Appl Environ Microbiol.* 2013;79:5728-5734.
20. Barker AK, Zellmer C, Tischendorf J, et al. On the hands of patients with *Clostridium difficile:* a study of spore prevalence and the direct effect of hand hygiene on *C. difficile* removal. *Am J Infect Control.* 2017;45:1154-1156.
21. Bobulsky GS, Al-Nassir WN, Riggs MM, Sethi AK, Donskey CJ. *Clostridium difficile* skin contamination and environmental shedding in patients with *C. difficile* - associated disease. *Clin Infect Dis.* 2008;46:447-450.
22. Sethi AK, Al-Nassir WN, Nerandzic MM, Bobulsky GS, Donskey CJ. Persistence of skin contamination and environmental shedding of *Clostridium difficile* during and after treatment of *C. difficile* infection. *Infect Control Hosp Epidemiol* 2010;31:21–27.
23. McFarland LV, Mulligan ME, Kwok RY, Stamm WE. Nosocomial acquisition of *Clostridium difficile* infection. *N Engl J Med* 1989;320:204–210.

© 2020 Barker AK et al. *JAMA Network Open.*

24. Struelens MJ, Maas A, Nonhoff C, et al. Control of nosocomial transmission of *Clostridium difficile* based on sporadic case surveillance. *Am J Med* 1991;91:S138–44.
25. Eckstein BC, Adams DA, Eckstein EC, et al. Reduction of *Clostridium difficile* and vancomycin-resistant *Enterococcus* contamination of environmental surfaces after an intervention to improve cleaning methods. *BMC Infect Dis* 2007;7:61.
26. Faires MC, Pearl DL, Berke O, Reid-Smith RJ, Weese JS. The identification and epidemiology of methicillin-resistant *Staphylococcus aureus* and *Clostridium difficile* in patient rooms and the ward environment. *BMC Infect Dis* 2013;13:342.
27. Dubberke ER, Reske KA, Noble-Wang J, et al. Prevalence of *Clostridium difficile* environmental contamination and strain variability in multiple health care facilities. *Am J Infect Control* 2007;35:315–8.

© 2020 Barker AK et al. *JAMA Network Open.*
